# Extraction Kinetics of As(V) by Aliquat-336 Using Asymmetric PVDF Hollow-Fiber Membrane Contactors

**DOI:** 10.3390/membranes8030053

**Published:** 2018-08-02

**Authors:** Said Bey, Hassina Semghouni, Alessandra Criscuoli, Mohamed Benamor, Enrico Drioli, Alberto Figoli

**Affiliations:** 1 Laboratoire des Procédés Membranaires et des Techniques de Séparation et de Récupération, Faculté de Technologie, Université de Bejaia, Béjaïa 06000, Algérie; mohamedbenamor@yahoo.fr; 2 Institute on Membrane Technology (ITM-CNR), Via P.Bucci 17/C, 87030 Rende (CS), Italy; semghounihassina@gmail.com (H.S.); a.criscuoli@itm.cnr.it (A.C.); e.drioli@itm.cnr.it (E.D.)

**Keywords:** As(V), membrane contactor, Aliquat-336

## Abstract

This work focuses on the study of the mass transfer of arsenic(V) through asymmetric polyvinylidene fluoride hollow-fiber membrane contactors using Aliquat-336 as an extractant. In the first part of this work, the fibers were prepared and characterized by SEM and by determining their thickness and porosity. From SEM pictures, an asymmetric structure was obtained that was characterized by an inner sponge-like structure and outer finger-like structure with a pore radius and porosity about 0.11 µm and 80%, respectively. In the second part, the prepared fibers were used as membrane contactors for the study of mass transfer of arsenic(V), investigating the effect of several parameters such as pH, temperature, and initial concentration of the feed. The overall mass transfer coefficient of As(V) was around 6 × 10^–6^ cm/s.

## 1. Introduction

Arsenic, a highly toxic element, can be found in natural water system in its two main forms, organic and inorganic. Generally, inorganic arsenic has two different oxidation states in aqueous solution: trivalent and pentavalent [1]. It is introduced into the human body through drinking water and food, causing lung, liver, kidney, and skin cancer. Its consumption also leads to disturbance of cardiovascular and nervous system function, which eventually leads to death [1,2]. The presence of arsenic in nature is due to its abundance in the Earth’s crust and as a result of pyro-metallurgical and hydrometallurgical activities. Moreover, arsenic was used for wood preservation, as anti-fungicide in agriculture, as additives for lead car batteries, and in semiconductor industries. A rich review on arsenic in metallurgy was reported by Nazari et al. [3].

Up to now, several techniques were developed for arsenic removal, such as precipitation, ion exchange, adsorption, membrane process, and solvent extraction [1,4,5,6,7,8,9,10,11,12]. In the literature, the most employed adsorbents for arsenic removal were reported in a critical review by Mohan et al. [10]. However, these systems present many drawbacks in terms of cost and regeneration of the activate materials. In the last two decades, liquid membranes gained an important interest for arsenic removal from aqueous solution. Perez et al. [9] studied As(V) transport through a supported liquid membrane impregnated with Cyanex-921. They reported that more than 94% of As(V) was transported in two hours. Hollow fiber–supported liquid membrane using Cynaex-923, tri-nbutyl phosphate (TBP), Cyanex-301, trioctyl amine (TOA), and Aliquat-336 as extractant was used in As(V) transport. In this study, Panchroen et al. [13] reported the transport of As(V) from produced water in the gas separation plant in the Gulf of Thailand with efficiency depending on the type of extractant. They reported that Aliquat-336 at 35% (*v*/*v*) allows a high percentage of extraction of As(V). Güell et al. [14] focused their investigation on the transport of As(V) through a supported liquid membrane containing Aliquat-336 as a mobile carrier and compared its performance with that of an anion exchange membrane. They reported the effective transport of As(V) by the two systems. In our previous work [15], asymmetric hollow-fiber membrane contactors based on PVDF using Aliquat-336 in kerosene were employed. From the experimental tests, the extraction of As(V) was effective with an efficiency of 70%. Recently, Srivastava et al. [16] investigated the extraction of As(V) by emulsion liquid membrane using Aliquat-336 and 2-ethyl hexanol. From the results, 2-ethyl hexanol is a better carrier for As(V) removal from aqueous solution at low pH. In the literature, a review on the extraction of arsenic by liquid membrane was reported by Marino and Figoli [17]. Table 1 summarizes the most important research on Arsenic removal by liquid membranes.

With respect to liquid membranes that suffer from the problem of organic loss and consequent reduced membrane stability, membrane contactors present a longer life-time during liquid-liquid extractions. For arsenic removal, Aliquat-336 has often been used in the organic phase in both types of membrane operations, as reported in Table 1. Therefore, considering the positive results obtained in our previous work with this extractant [15], a deeper analysis of the system in terms of overall mass transfer coefficient is presented in this paper. In particular, this work focuses on the extraction kinetics of As(V) by Aliquat-336 using hollow-fiber membrane contactors.

## 2. Experimental

### 2.1. Chemicals

Methyl trioctylammonium chloride (Aliquat-336) (Sigma-Aldrich, Darmstadt, Germany) was used as extractant. The diluent was analytical-grade kerosene (Sigma-Aldrich) used without further purification. PVDF Solef^®^6012 polymer powder was kindly provided by Solvay Specialty Polymer (Bollate, Italy) and used for the preparation of the hollow-fiber membranes.

### 2.2. Preparation of PVDF Microporous Hollow-Fiber (HF) Membranes by Dry-Wet Spinning Process

In the first part of the experimental study, hydrophobic PVDF hollow-fiber membranes have been produced by dry-wet spinning process. The polymeric solution was doped with water and polyvinyl pyrrolidone, PVP (k17), a modifier (BASF, Ludwigshafen, Germany), and dimethyl formamide (DMF) was used as solvent. Bore liquid consisted of DMF and water at 25/75 (*v*/*v*). They were heated and pumped through the inner tube of the spinneret with the flow rate of 20 mL/min. The experimental set up of hollow-fiber membranes preparation is shown in our previous work [15].The structural and permeation-related parameters of the prepared hydrophobic hollow-fiber membranes were characterized by means of dimensional and performance factors. Among dimensional parameters, outside (d_o_) and inside diameters (d_i_) and the thickness (δ) of the fibers were measured. Pore size, porosity, and pore radius also were measured. Moreover, the porous structures of the membranes were documented by the scanning electron microscopy (SEM) pictures.

### 2.3. Non-Dispersed Solvent Extraction of As(V) by Aliquat-336

**Figure 1:**
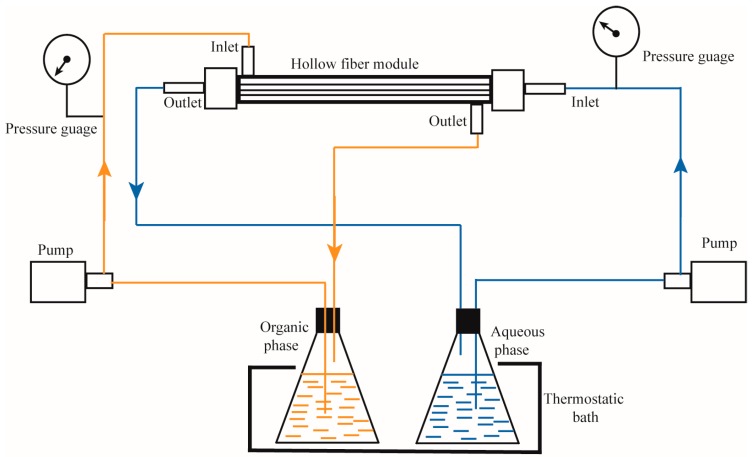
Hollow-fiber membrane contactors set up.

The non-dispersed solvent extraction experiments were carried out using a lab-made module based on PVDF asymmetric hollow fibers prepared by immersion-precipitation. The organic phases were prepared by dissolving Aliquat-336 in kerosene modified by 4% of octanol to avoid any phase segregation. The feeding solution of arsenic was prepared by dissolving a certain quantity of NaHAsO_4_ in bi-distilled water. The aqueous feed phase flowed in the lumen side and the organic in the shell side. In this case, since the organic phase wets spontaneously the membrane, a positive static pressure on the aqueous feed phase was applied, to prevent the organic from dispersion into the aqueous feed phase to form an emulsion. The experiments were carried in recirculating mode and samples of 1 mL were taken from the reservoir. The volumes of organic and aqueous phases were 175 mL and 300 mL respectively. Each experiment was repeated at least two times. The maximum error was ±2%. The concentration of arsenic ions was determined by inductivity coupled plasma spectroscopy. Figure 1 shows the scheme of the hollow-fiber membrane contactors set up.

The overall mass transfer coefficients *K* was calculated from the following equation, obtained from a mass balance on the system [19,20].

(1) K=−d·v4·L1(1+(QinH·Qout))·ln[1+Vin·((1Qin)+(1Qout))1+(VinH·Vout)·ln(ΔCΔC0)t]

where

(2) lnΔCΔC0=lnCin(1+(VinH·Vout))−Cin0((VinH·Vout)−(Cout0H))Cin0−(Cout0H)

where

*d*: inside hollow fiber diameter;
*v*: aqueous flow velocity;
*L*: length of the fiber;
*H*: partition coefficient;
$Q_{in}$ and $Q_{out}$ are the aqueous and the organic flow rates, respectively;
$V_{in}$ and $V_{out}$ are the aqueous and the organic reservoir volumes, respectively;
$C_{in}$, $C_{in}^{0}$ and $C_{out}^{o}$ are the aqueous feed concentration at time *t*, the initial aqueous feed concentration and initial organic concentration, respectively.

In the extraction of As(V), the form of the species in water is important, especially when an ion exchange process was used. In our case, a basic extractant, Aliquat-336 diluted in kerosene, was used. The basic equilibrium and effect of the pH, in the range of 0.87 to 12.5, were studied. The reaction involved in the extraction is an ion exchange between Aliquat-336 and the oxyanions of As(V), depending on pH of the aqueous solution and the forms of the oxyanions. It seems that the divalent and monovalent oxyanions of As(V) present in the pH-range 6.98 to 8.2 are favorable for extraction by Aliquat-336 according to the equations below.

(3) $$\bar{\left(R_{3}{\mathrm{NH}}^{+},{\mathrm{Cl}}^{-}\right)}+H_{2}{\mathrm{SO}}_{4}^{-}\Leftrightarrow \bar{\left(R_{3}{\mathrm{NH}}^{+},{\;H}_{2}{\mathrm{SO}}_{4}^{-}\right)}+{\mathrm{Cl}}^{-}$$

(4) $$\bar{2\left(R_{3}{\mathrm{NH}}^{+},{\mathrm{Cl}}^{-}\right)}+H_{2}{\mathrm{AsO}}_{4}^{2-}\Leftrightarrow \bar{{\left(R_{3}\mathrm{NH}\right)}_{2}{\mathrm{HAsO}}_{4}}+2{\mathrm{Cl}}^{-}$$

## 3. Results and Discussion

### 3.1. SEM Analysis

**Figure 2:**
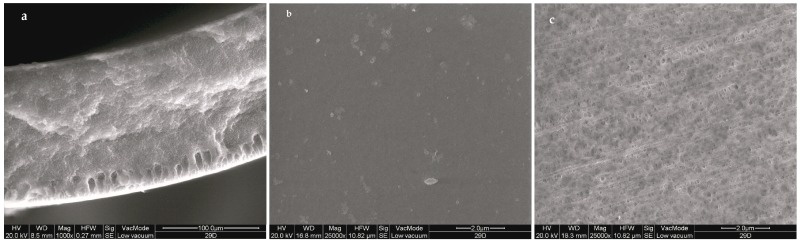
Scanning electron microscopy (SEM) pictures of the cross-section (**a**), the outer (**b**), and inner (**c**) surfaces of hollow fibers.

SEM pictures show an asymmetric structure characterized by a large inner sponge structure and a thin finger-like structure at the outer surface. The asymmetry of the fiber can be appreciated by looking inner and outer surfaces of the hollow fibers that are porous and smooth, respectively (Figure 2).

Table 2 summarizes the properties of the modules used in this study.

### 3.2. Solvent Extraction Studies of As(V) by Means of Hollow-Fiber Membrane Contactors

**Figure 3:**
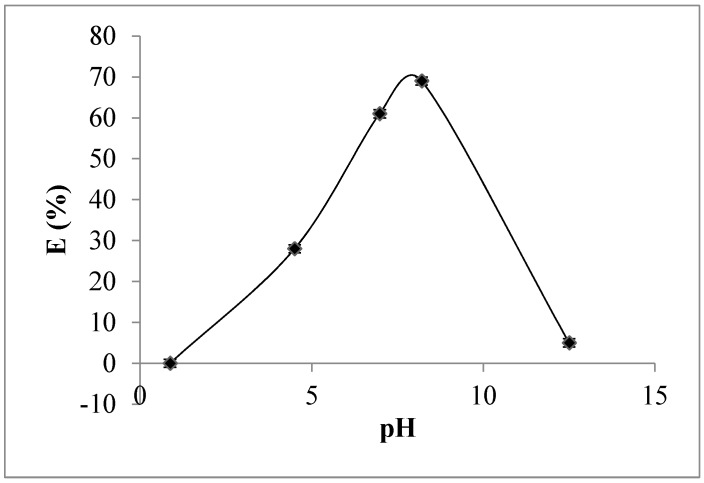
Equilibrium extraction of As(V) by Aliquat-336. Aqueous phase: [As(V)] = 60 ppm, flow-rate: 0.47 mL/s. Organic phase: [Aliquat-336] = 30% (*v*/*v*) + Kerosene + 4% (*v*/*v*) octanol; flow-rate: 1.4 mL/s; Membrane: PVDF hollow fiber, T = 25 °C. ∆P = 0.3 bar.

The basic equilibrium and mass transfer experiments produced the following results. Figure 3 shows the extraction of As(V) into 30% of Aliquat-336, as function of the pH. The reaction involved in the extraction is an ion exchange type (Equations (3) and (4)). Cl^−^ ions in Aliquat-336 are replaced by the oxyanions of As(V). Since the predominance of monovalent and divalent oxyanions of As(V) is for pH values from 6.98 to 8.2, the high percentage of extraction is obtained in this pH range, reaching 70%. By decreasing the pH to 4.5 the partition coefficient decreases, due probably to the formation of neutral species which cannot be extracted by Aliquat-336. At very low pH (close to zero) the neutral species are predominant and the extraction is very low. Furthermore, in basic medium at pH = 12.5 no extraction of arsenate was observed. Consequently, trivalent oxyanions of As(V) cannot be extracted by Aliquat-336. Accordingly, the rest of experiments were carried out with 30% of Aliquat-336 diluted in kerosene and initial aqueous pH of 6.98.

**Figure 4:**
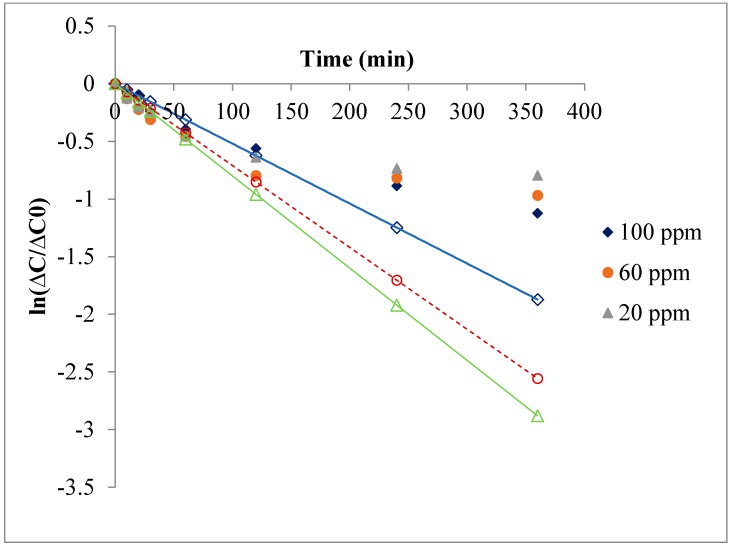
As(V) concentration vs time. Aqueous phase: pH = 6.98; Flow-rate: 0.47 mL/s. Organic phase: [Aliquat-336] = 30% (*v*/*v*) + Kerosene + 4% (*v*/*v*) octanol; flow-rate: 1.4 mL/s; Membrane: PVDF hollow fiber, T = 25 °C. ∆P = 0.3 bar.

In the hollow fiber experiments, the concentration of As(V) varies semi-logarithmically with time, as shown in Figure 4. In particular, the logarithm of the concentration difference is linear in time at the beginning of the experiment. This means that the data are consistent with Equation (1) and (2) at high metal concentration. Consequently, at short experiment time, the overall mass transfer coefficient from the initial slope of these data can be calculated. This overall mass transfer coefficient shows the behaviour of As(V) extraction in this system. For example, as reported in Table 3, it varies significantly with the initial concentration and pH of feed solution but weakly with temperature. In particular, the overall mass transfer coefficient increases by increasing the initial concentration of As(V), reaching the value of 7 × 10^–6^ cm/s at 100 ppm. The result obtained at the high temperature of 50 °C could be explained by the presence of PVP in hollow fiber, which reduces the contact area between the two phases, or by the exothermic reaction of As(V) with the extractant.

## 4. Conclusions

In this study, asymmetric hollow-fiber membranes were prepared by dry wet phase inversion and applied to extraction of As(V) by Aliquat-336 in hollow-fiber membrane contactors system under several conditions. Arsenic was extracted in its two mainly forms species such as monovalent and divalent by Aliquat-336. However, the trivalent forms in basic medium were not extracted. No effect of temperature on the kinetic extraction of As(V) was observed. The kinetic extraction was, on the contrary, affected by the initial feed concentration and pH.

The mass transfer of As(V) through asymmetric PVDF hollow fibers was around 6 × 10^−6^ cm/s, comparable to other metal ions-extractant/membrane contactors reported in the literature.

## Figures and Tables

**Table 1:** Removal of arsenic by liquid membrane systems.

Liquid Membranes	Feed Phase	Organic Phase	Membrane	Stripping Phase	Ref.
SLM	As(V) [H_2_SO_4_] = 2 M	Cyanex-921	PVDF-millipore GVHPO4700	[Na_2_SO_4_] = 1 M	[9]
HF SLM	Produced water in gas plant [As] = 1.2842 ppm	Cyanex-923; Cyanex-301 TOA; Aliquat-336	Celgard-X-30240-PP HF	[NaOH] = 0.5 M	[13]
SLM	[As] = 10 ppm	Aliquat-336: 0.5 M	PVDF Durapore	[NaCl] = 0.1 M	[14]
PIM	[As(V)] = 3000 ppm [H_2_SO_4_] = 2 M	DBBP	Cellulose tri-acetate	[LiCl] = 2 M	[18]
Membrane contactors	[As] = 50 ppm	Aliquat-336	PVDF asymmetric hollow fiber	-	[15]
ELM	[As] = 100 ppm	Aliquat-336 + Span 80 2-ethyl hexanol + Span 80	-	[NaOH] = 0.5 M	[16]

**Table 2:** Properties of the hollow-fiber membrane contactors module.

Properties	Description
Material	PVDF
Hollow fiber inner diameter (mm)	1.35
Pore size (µm)	0.11
Number of fibers	3
Porosity (%)	80
Surface (cm^2^)	22.9
Module diameter (cm)	1
Module length (cm)	18

**Table 3:** Overall mass transfer coefficients of As(V).

Parameters		*K* × 10^6^ (cm/s)
pH	12.5	0
	8.2	7.54% ± 2%
	6.98	6.05% ± 2%
	4.5	2.11% ± 1%
Initial As(V) concentration (ppm)	100	7.04% ± 3%
	60	6.05% ± 2%
	20	4.96% ± 1.5%
Temperature (°C)	25	6.05% ± 2%
	40	6.45% ± 2.5%
	50	3.45% ± 1%
